# Sex bias in multiple sclerosis and neuromyelitis optica spectrum disorders: How it influences clinical course, MRI parameters and prognosis

**DOI:** 10.3389/fimmu.2022.933415

**Published:** 2022-08-09

**Authors:** Petra Nytrova, Ondrej Dolezal

**Affiliations:** ^1^ Department of Neurology and Centre of Clinical Neuroscience, First Faculty of Medicine, Charles University in Prague and General University Hospital, Prague, Czechia; ^2^ Department of Neurology, Dumfries and Galloway Royal Infirmary, NHS Scotland, Dumfries, United Kingdom

**Keywords:** multiple sclerosis, neuromyelitis optica spectrum disorders, sex bias, pregnancy, magnetic resonance imaging, brain atrophy, disease progression

## Abstract

This review is a condensed summary of representative articles addressing the sex/gender bias in multiple sclerosis (MS) and neuromyelitis optica spectrum disorders (NMOSD). The strong effects of sex on the incidence and possibly also the activity and progression of these disorders should be implemented in the evaluation of any phase of clinical research and also in treatment choice consideration in clinical practice and evaluation of MRI parameters. Some relationships between clinical variables and gender still remain elusive but with further understanding of sex/gender-related differences, we should be able to provide appropriate patient-centered care and research.

## Introduction

The predominance of females among patients with autoimmune central nervous system disorders such as multiple sclerosis (MS) and neuromyelitis optica spectrum disorders (NMOSD) is well recognized. Several sex-specific factors, including sex hormones themselves and genetics - the presence of two X chromosomes versus one X and one Y chromosome, and environmental and societal factors including dietetic habits might play an important role in susceptibility and manifestation of autoimmune disorders (1–3). Furthermore, these factors can influence each other in the interconnected functional network. In this review, we discuss current views on sex bias in MS and NMOSD and their impact on disease course, prognosis, and MRI findings.

Previous research naturally focused on the influence of sex hormones, but it seems that hormonal variances between sexes explain clinical differences only to some extent as female sex bias is frequently observed even in autoimmune diseases with onset in childhood when estrogen levels do not differ between sexes, or in postmenopausal women (4). A possible explanation for these differences could be hidden in sex chromosomes, which were studied on animal models of different autoimmune disorders (5, 6). Several X chromosome genes are known to be involved in immune responses (7), one of which is Forkhead box p3 (Foxp3) (5). This gene is important for the development and function of CD4+CD25hi T regulatory cells (Treg) (8, 9), which might contribute to the relative resistance to experimental autoimmune encephalomyelitis in males (10). Foxp3 expression during the induction of Treg function is controlled by epigenetic mechanisms at the transcriptional level that involve Foxp3 DNA methylation (11, 12). Furthermore, there are not only X-linked genes that could influence the sex bias but also X-linked control mechanisms like non-coding microRNA (miRNA), which is involved in the regulation of gene expression by suppressing mRNA translation or triggering mRNA degradation (13–15). The upregulation of X-linked miR-18 during relapse in patients with MS was described (16). The reason for the absence of miRNA in the Y chromosome is unknown (14).

## Sex bias in epidemiology and pathophysiology of MS and NMOSD

MS is an acquired inflammatory demyelinating disorder predominantly affecting young females in 2-3:1 female to male (F:M) ratio for relapsing MS in developed countries (17–19). Furthermore, several studies have shown that multiple sclerosis F:M ratio of cases increases over time when serial cross-sectional comparisons were made (17). In contrast, primary progressive MS affects men and women equally (20, 21). Previously, the cellular immunology of relapsing multiple sclerosis was considered to be principally T-cell driven. However, recent research revealed that autoimmune pathological processes in MS are more complex and involve multiple cell types and their functionally distinct subsets. Particularly in relapsing multiple sclerosis pathological mechanisms involve imbalanced interactions between T cells, myeloid cells, B cells, and their effector and regulatory subpopulations (22). There is likely no qualitative difference in the pathology between relapsing and progressive MS and to some extent including primary progressive MS. However, the contribution of the pathological processes and alterations differs quantitatively. Focal new and active white matter lesions (representing inflammation) are most numerous in early (acute and relapsing) MS and lesional volume changes are of less dominance when patients enter the progressive stage (23). Diffuse changes in the normal-appearing white matter are sparse in early MS but very pronounced in patients with progressive MS (24). These changes eventually lead to localized (e.g. cortical) and global brain atrophy which can be seen on brain MRI. Therefore, the most commonly used MRI marker for monitoring inflammatory activity is the number or volume of MRI hyperintense lesions (on T2 weighted or FLAIR images). Modern techniques can successfully detect cortical lesions as well (25). Neuromyelitis optica spectrum disorders are rare inflammatory disorders of the central nervous system, manifesting clinically as optic neuritis, myelitis, and certain brain and brainstem syndromes (26). NMOSD may include aquaporin 4 (AQP4)-antibody seropositive autoimmune astrocytopathic disease and AQP4-antibody seronegative patients as well (27). A part of those seronegative patients with clinical NMO phenotype have antibodies to myelin oligodendrocyte glycoprotein (MOG) (28, 29) and represents a relatively new disease entity called myelin oligodendrocyte glycoprotein-antibody associated disease (MOGAD) (30, 31). AQP4-antibody seropositive NMOSD has a high female to male ratio (up to 9:1) with later onset (at the average age above 40) compared to multiple sclerosis (32, 33).

## The effect of sex on the age of clinical onset and diseases course in MS and NMOSD

The relationship between age of onset and sex ratio in different life periods can help to explain the role of sex hormones in MS and NMOSD disease pathogenesis. Sex hormones can affect the function of the immune cells directly *via* binding to the steroid receptors and have various effects on cells of both the adaptive and innate immune systems (3, 34–37). Relapsing MS and NMOSD can sometimes manifest in children and adolescents as well, although rarely. It can be difficult to differentiate MS from other inflammatory demyelinating diseases at an early age. Multiple sclerosis presents with its typical female predominance from puberty onwards, corresponding with reproductive maturing, whilst males seem to be over-represented at very young ages (38). It seems that within the relapsing MS group there are sex differences in relapse characteristics and in the extent of recovery where males show more incomplete recovery from a relapse and more persistent disability (traditionally represented by the Expanded Disability Status Scale, EDSS) (39–41). These sex differences in disability were not observed in late-onset MS or in primary progressive form (41, 42). Kalincik et al. showed that women tend to present with visual and sensory relapses more frequently than men, who are relatively more likely to present with pyramidal (motor), brainstem, and cerebellar relapses (43). Although several studies have evaluated the effect of menopause on MS disease course, including relapse rates, disability progression, and patient-reported outcomes. Data are inconclusive so far but might indicate some increase in disability when comparing before and after menopause stages (44–46). A systemic hormone treatment used in postmenopausal MS patients was associated with the better physical quality of life in postmenopausal women (47). The effect of hormone therapy (estriol or estroprogestins) combined with glatiramer acetate or interferon beta was also analysed in clinical trials in women with relapsing MS (48–50).

Relatively little is also known about transgender (TGD) issues in patients with multiple sclerosis, who face substantial challenges stemming from chronic illness in combination with psychosocial and other health factors related to transgender issues (51). Gender-affirming exogenous hormone use must be considered because it can influence the risk of MS. The main pattern of treatment for TGD female to male (TrM) is lifelong testosterone (52) and for TGD male to female (TrW), oral or transdermal estrogens, progesterone, and an antiandrogen (cyproterone acetate) are used (53). Pakpoor et al. provided some evidence supporting a potential role for low testosterone and/or feminising hormones on MS risk in TGD males to females (54).

The female predominance in NMOSD occurring in children and adolescents is seen at the ratio of 1.5:1 and 3.25:1 respectively (55). The other study has shown a 5:1 F:M ratio of AQP4-antibody seropositive patients younger than 12 years (56). This being said, elderly individuals are also at risk of developing NMOSD. The proportion of AQP4-antibody seropositive individuals (detection rate), defined by a decade of age, increased exponentially in women after the age of 50. This was not observed in men of the same age (57). How menopause may affect the age of manifestation of NMOSD and the role of sex hormones has not been studied in detail. Increasing age was associated with a decreased risk of relapse in AQP4-antibody seropositive patients (58). Some patients with typical clinical manifestations for neuromyelitis optica are consistently seronegative for AQP4-IgG. The French and German studies and Mayo group reported almost equal or slightly increased F:M ratio (1.2:1; 1.9:1; respectively 1:1) in these cohorts when Wingerchuk criteria for NMO from 2006 were applied (59–61). The proportion of seropositive MOG-IgG patients with NMO phenotype varies between different studies based on applied diagnostic criteria and sensitivity of the cell-based assay used for the antibody assessment. MOG-antibody seropositive patients can account for about 40% of AQP4-antibody seropositive patients who were diagnosed according to the 2015 International panel on NMOSD diagnosis when the highly sensitive live cell-based assay was used (62). The clinical manifestation of MOGAD differs between age groups. The most common presentation in children is acute disseminated encephalomyelitis (ADEM) compared to adults, who typically suffer from optic neuritis at the onset. In the youngest cohort (age <10 years) of MOGAD, we cannot see much difference between males and females but there is a slight female predominance in adolescents and adults (63). Kim et al. have shown an impact of sex on disease onset age and site of relapse when AQP4-antibody seropositive male NMOSD patients had a higher age at onset than women and were less likely to develop optic neuritis as the initial symptom (64). Kitley et al. described a UK-Japanese cohort of patients with disease onset < 30 years of age in which 61% of patients first presented with optic neuritis compared with only 18% presenting with longitudinally extensive transverse myelitis (LETM). In older groups (50 years of age) we see almost the opposite picture as 66% presented with LETM compared with 28% presenting with optic neuritis (65). Whether sex hormones might influence (directly or indirectly) a development or severity of optic neuritis and protect the spinal cord remains unanswered. On the other hand, the protective effects of sex hormones on remyelination after optic neuritis were studied in several works (66–68).

## Radiological aspects of sex difference in MS and NMOSD

**Figure 1:**
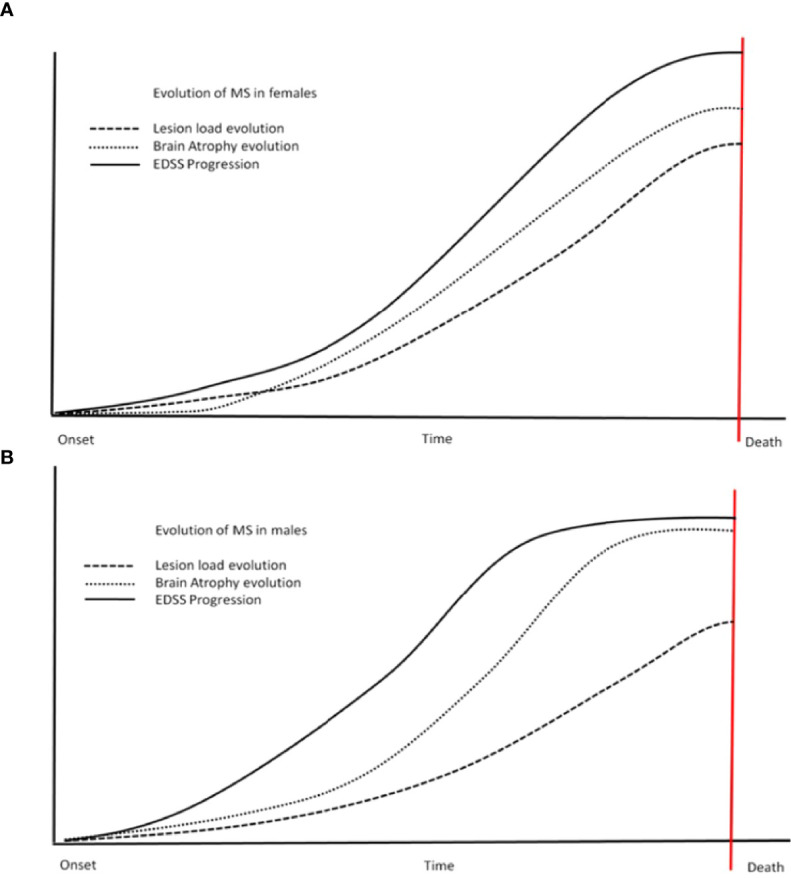
Steeper progress for males? This schematic diagram shows the differences in different variables between the sexes (females - **A**, males - **B**). Steeper changes are more obvious in males **(B)** - including atrophy and EDSS. Males also reach a plateau of variables sooner than females (the scheme is not to scale regarding time and values, reflecting trends only). EDSS, expanded disability status scale.

Brain atrophy, including grey matter and white matter atrophy measurement, is recently becoming a routine marker to monitor the disease in clinical studies and clinical practice. Over the last 20 years, different studies reported significant differences between sexes in variable measures. Generally, it seems that males are showing more, traditionally associated with degenerative processes, grey matter pathology, and atrophy (69). It seems that grey matter atrophy is affecting not only cortical regions but also deep grey matter represented by the reduction of neuronal mass in basal ganglia (putamen) and thalamus resulting in impairment of cognitive functions (70). Therefore, you can find a very different extent of atrophy in male and female patients with almost identical clinical histories. The brain atrophy dominant in males has been reported in groups of different ethnic origins (71). These sex-specific differences in atrophy measures are seemingly not as prominent early in the disease (72), but changes in those variables, however discreet, are likely pre-dating changes in the clinical picture (73). More questionable results were obtained while studying lesion volume/lesion load in MS patients (74, 75). This would not be surprising as lesion load varies significantly between individuals irrespective of gender. See schematic diagram (**Figure 1**) summarizing theoretical differences of sex bias in lesion volume, EDSS (clinical scale), and brain atrophy.

Lesions, predominantly present in white matter, would have an impact on white matter (WM) volume too. This obvious relationship between white matter lesions and white matter volume would explain why studies looking at white matter atrophy are showing more contradictory findings. In some studies, it seems that males show more prominent white matter changes associated with axonal loss than females (76). In other work, WM atrophy was even more prominent in females (69). Atrophy of all compartments is seen even in the early stages of PPMS (77). Artificial intelligence approaches have been recently tried to evaluate future risks, estimate disability progression, and most importantly monitor response to medication (atrophy-led v. lesion-led estimation) (78).

Unfortunately, no representative studies are focusing on MRI differences between sexes in NMOSD nor the impact of pregnancy on MRI parameters. In recent decades research paid attention to the role of iron and its metabolism in MS and NMOSD. Brain iron homeostasis is known to be disturbed in multiple sclerosis (79–81). The progression of disability in MS seems to inversely correlate with iron concentration, especially in a deep grey matter on MRI imaging (quantitative susceptibility mapping), which could have prognostic and diagnostic value (e.g., helping to differentiate between relapsing or primary progressive MS and in AQP4-antibody seropositive NMOSD) (82–84). The relationship between the clinical stage of MS, disease progression, and amount of iron differs between brain structures examined (putamen, caudate, inflammatory lesions, thalamus, normal-appearing white matter, etc.) (82–86). A correlation was found between the disability (EDSS) and magnetic susceptibility in the putamen in remitting MS (84). However, it is unclear if iron concentration changes are instead related to atrophy and loss of structure with lower concentrations of iron (e.g. myelin and calcium-rich structures) (85). Dedicated research looking at iron levels and sex differences is still to be done as many conducted studies did not analyse that relationship.

## Disease severity during pregnancy and the postpartum period in MS and NMOSD

The influence of sex hormones on autoimmune diseases including the changes in disease severity and activity during or after pregnancy has been reported in many autoimmune disorders such as systemic lupus erythematosus, myasthenia gravis, etc. (87, 88). Since high levels of hormones during pregnancy enhance Th2 response, this may suppress MS which is driven by Th1 response (89). Pregnancy is not associated with an increased risk of a flare of disease activity in MS. On the contrary, during the post-partum period lesion volume and inflammatory activity can increase T1 lesion volume “black holes” as well as T2 lesion volume in MS. It is usually followed by the clinical activity of the disease (90). While short-term consequences of pregnancy in MS are deemed proven it remains contentious what impact this has on brain atrophy and disability progression in the long term (91–93). Assisted reproductive techniques using gonadotropin-releasing hormone analogues (GnRH; either agonists or antagonists) might be associated with clinical (increased annualized relapse rate during the 3 months following *in vitro* fertilisation) and MRI visible inflammatory activity in MS (94–96). The administration of GnRH antagonist over agonist mainly in females <40 years of age is preferred (97, 98).

Less known is about NMO and pregnancy. NMOSD is mediated mostly by Th2 lymphocytes therefore a higher risk of relapse can be expected. Women with NMO also have an elevated rate of pregnancy complications including preeclampsia, which are associated with increased Th17 cells and reduction of T-regulatory cells (99). These in turn can enhance inflammation in NMOSD and be associated with increased relapse rates and disability in patients with NMOSD during pregnancy, and especially in the early postpartum period (100–102). Increased risk of relapse in those periods in NMOSD patients is also associated with discontinued or insufficient immunosuppressive treatment (101). Pregnancy complications in AQP4-autoantibody seropositive patients might be also related to other autoimmune comorbidity or the presence of autoantibodies such as antiphospholipid antibodies, which have been described in combination (or in absence) of SLE in NMOSD patients (103–106). Aquaporin-4 is expressed by the human placenta (107) and it has been demonstrated that AQP4-IgG could be a causative agent in increased miscarriages in females with AQP4-antibody seropositive NMOSD (105, 108, 109).

Although pregnancy in MS patients is not associated with increased disease activity as mentioned above, it is necessary to consider the disease activity before pregnancy, especially the type of therapy. One of the aspects that must be considered during the reproductive age of MS patients is the teratogenicity of the disease-modifying therapies. Teriflunomide is classified as a teratogen of category X (for both females and males), therefore expected benefits from this treatment do not outweigh drug-associated risks, and its use in pregnant women is contraindicated (110). There was so far no evidence of increased rates of spontaneous abortion, decreased birth weight or congenital malformation in human trials or retrospective pharmacovigilance observation (111, 112). Teriflunomide plasma levels of less than 0.02 mg/L are expected to have no teratogenic impact (112), therefore the rapid elimination procedure of teriflunomide in case of pregnancy is recommended. Another important aspect of pregnancy planning in MS patients is to consider discontinuation of highly effective therapies such as fingolimod or natalizumab. It has been reported that stopping fingolimod and natalizumab may be a cause of worsening neurological status (113). Disease reactivation following fingolimod cessation is more common in younger patients, those with greater disease activity before cessation, and those who switch to a low-efficacy therapy (114). Fingolimod discontinuation could be a cause of life-threatening relapse, although this is a rare situation (115). Saying all this we have to bear in mind that fingolimod is teratogenic in animals, therefore, would not be a suitable treatment in pregnancy contrary to natalizumab which can be used until the 34th week of gestation in the case of patients with high disease activity (116). Neurologists and obstetricians must be aware of the potential complications of a pregnancy in a woman who has MS but specifically NMOSD.

## Summary

As seen above, sex bias is an extremely important factor (summarized in **Table 1**). In many cases it defines the prognosis and fate of individual patients. Current up-to-date research is helping us to understand the relationships between the pathophysiology of MS and NMOSD and gender stands in three main areas: clinical (experience of treating clinicians); immuno-chemical (basic and applied research); and radiographic (MRI studies, volumetry, etc.). The key to understanding is a multidisciplinary approach covering all these areas. Sex/gender effect on the incidence, activity, and progression of these disorders should be implemented in the evaluation of any phase of clinical research and treatment choice consideration in clinical practice and evaluation of MRI parameters. Some relationships between clinical variables and sexes remain elusive but with further understanding of sex/gender related differences, we should be able to provide appropriate patient-centered care and research.

**Table 1:** Summary of sex bias in relapsing multiple sclerosis (MS) and AQP4-antibody seropositive neuromyelitis optica spectrum disorders (NMOSD).

	Relapsing MS	AQP4-IgG^pos^NMOSD	Possible explanation/association
Epidemiology (female to male ratio)	2-3:1 in adults (17–19) women show earlier onset (117)	up to 9:1 in adults (32, 33) up to 5:1 in children younger < 12 years (56)	sex hormones affect directly or indirectly function of immune cells ; X dosage compensation and escape from X-inactivation; imprinting of X chromosome genes; epigenetics; X-linked non-coding microRNA (1–16);
Clinical features	visual and sensory relapses more frequent in women; motor, brainstem, and cerebellar relapses more frequent in men (43)	male patients have higher age at onset and are more likely to develop myelitis as a first symptom (64)	unknown
Imaging	GM and central atrophy are more advanced in male patients, whereas lesion load or gadolinium enhancing lesions are more advanced in female patients (69, 70, 118)	unknown	men develop a lower number of inflammatory lesions in the CNS, but a higher number of degenerative lesions with extensive axonal loss; males have a higher incidence of cortical GM lesions compared to females (120)
Disability progression	males show more incomplete recovery from a relapse and more persistent disability (119)	probably not related to sex; influenced by age of disease onset and by delay in diagnosis/treatment (64)	absence of protective effects of females hormones; Y gene presence or absence;differences in parental X imprinting of X chromosome genes (1–7) – f.e. a different expression of TLR7 by cortical neurons in males (121) is also considered in MS
Risk of relapse	higher in women (119)	not studied but the risk of relapse is more likely to be associated with younger age(64)	effects of females hormones on the immune system and other sex-related factors that can play role in higher susceptibility for MS in women (1–5)

GM, grey matter; WM, white matter; TLR7, toll-like receptor 7.

## Author contributions

PN has been involved in source collection and wrote the clinical and immunology section of the article. OD was involved in source collection and wrote the radiology section. Both authors participated equally in the graphic and clinical sections. All authors read and approved the final manuscript.

## Funding

The work was supported by the Czech Ministry of Education – project Cooperatio LF1, research area Neuroscience, by Czech Ministry of Health – project NU22-04-00193, and the project of National Institute for Neurological Research (Programme EXCELES, ID project No LX22NPO5107- funded by the European Union-Next Generation EU.

## Conflict of interest

PN has received speaker honoraria and consultant fees from Biogen, Novartis, Merck, Roche, and financial support for research activities from Roche and Merck. OD has received in the past funding for research, speaking honoraria, advisory boards, travel or educational support from Bristol-Myers Squibb, Sanofi Genzyme, Novartis, Biogen and Merck Serono pharmaceuticals.

## Publisher’s note

All claims expressed in this article are solely those of the authors and do not necessarily represent those of their affiliated organizations, or those of the publisher, the editors and the reviewers. Any product that may be evaluated in this article, or claim that may be made by its manufacturer, is not guaranteed or endorsed by the publisher.
